# Elasto-Kinematics and Instantaneous Invariants of Compliant Mechanisms Based on Flexure Hinges

**DOI:** 10.3390/mi14040783

**Published:** 2023-03-30

**Authors:** Christian Iandiorio, Pietro Salvini

**Affiliations:** Department of Enterprise Engineering, University of Rome “Tor Vergata”, Via del Politecnico 1, 00133 Rome, Italy; salvini@uniroma2.it

**Keywords:** compliant mechanisms, instantaneous invariants, MEMS, large displacements, nonlinear analysis

## Abstract

The kinematic synthesis of compliant mechanisms based on flexure hinges is not an easy task. A commonly used method is the equivalent rigid model, which involves replacing the flexure hinges with rigid bars connected with lumped hinges using the already known methods of synthesis. This way, albeit simpler, hides some interesting issues. This paper addresses the elasto-kinematics and instantaneous invariants of flexure hinges with a direct approach, making use of a nonlinear model to predict their behaviour. The differential equations that govern the nonlinear geometric response are given in a comprehensive form and are solved for flexure hinges with constant sections. The solution to the nonlinear model is then used to obtain an analytical description of two instantaneous invariants: the centre of instantaneous rotation (c.i.r.) and the inflection circle. The main result is that the c.i.r. evolution, namely the fixed polode, is not conservative but is loading-path dependent. Consequently, all other instantaneous invariants are loading-path dependent, and the property of instantaneous geometric invariants (independent of the motion time law) can no longer be used. This result is analytically and numerically evidenced. In other words, it is shown that a careful kinematic synthesis of compliant mechanisms cannot be addressed by only considering the kinematics as rigid mechanisms, and it is essential to take into consideration the applied loads and their histories.

## 1. Introduction

In the last two decades, compliant mechanisms [1,2,3] have produced a growing interest in academic and industrial fields [4,5]. These types of mechanisms manifest their motion through the deformation of some very slender parts [6,7,8] instead of kinematic pairs. Compliant mechanisms have some advantages when compared to lumped pairs: they do not require lubrication or maintenance as they have a monolithic form (directly replaceable if failure occurs); can be made via low-cost additive manufacturing; are not affected by clearance, friction and wear on contacting parts; and they can be very light. These features make them ideal for micro-electro-mechanical systems (MEMS) [9,10,11,12,13,14,15,16,17,18], micro-opto-electromechanical systems (MOEMS) [19,20], and precision engineering [21,22,23,24], including micro-manipulators [25,26,27,28] eventually driven by piezoelectric actuators (PEA) [29,30,31,32]. Other specific applications of compliant mechanisms include vibration isolation [33], precision polishing [34], micro-scribing [35] or micro-jetting [36].

On the other hand, the design of compliant mechanisms is tricky, as their motion involves large displacements/rotations [37,38,39,40,41] (i.e., highly nonlinear geometric behaviour) of the slender joints (flexure hinges). The design analysis requires a nonlinear structural approach. The main challenge regarding these mechanisms is finding consolidated methodologies to define the adequate sizing of flexible joints to realize the required trajectory (kinematic synthesis) and guarantee the desired fatigue life. Therefore, it is straightforward to observe that the design of compliant mechanisms must be addressed as a multi-objective problem.

The introduction of deformable bodies implies that compliant mechanisms do not depend on a countable number of degrees of freedom (dof), as is customary for rigid bodies; this dramatically increases the complexity of the design phase [42,43]. For this reason, in the literature, many authors have made use of pseudo-rigid models [2], in which the compliant behaviour is approximated (strictly for small movements around the reference configuration) using an equivalent rigid mechanism formed by ideal constraints [44,45]. This strategy aims to apply the standard methodologies of kinematic synthesis. Different studies have exploited this simplification, where the pseudo-rigid model has been used for various types of flexure hinges, including leaf [46], circular [47], parabolic [48] and notched [49]. The result is that lumped hinges and flexural springs replace the overall flexural hinges (the more lumped hinges and springs are used, the more the accuracy increases). Their locations are a function of the geometry of the compliant mechanism; however, they are also affected by the applied load directions and intensities. The shift of the centre of instantaneous rotation in the pseudo-rigid body model is usually not considered [50]. With the aim of improving this aspect, the motion of the centre of instantaneous rotation has been studied for flexure hinges only loaded by concentrated moments at the end [48,51,52].

**Figure 1:**
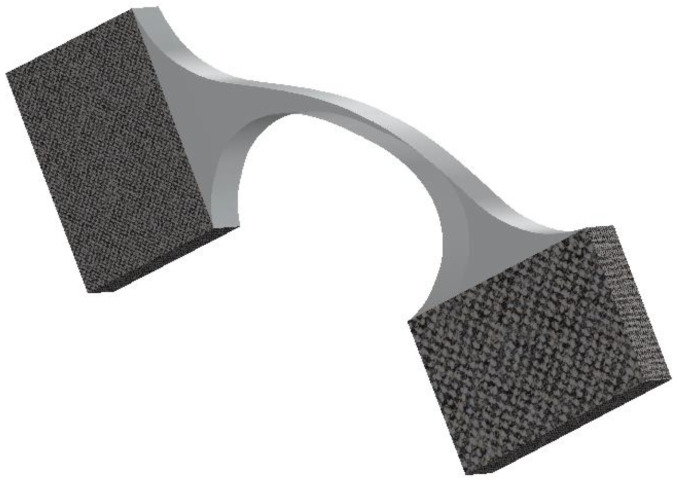
Connection of two rigid bodies through a flexure hinge.

With the purpose of providing a complete study of the instantaneous invariants of rigid bodies connected with flexure hinges (Figure 1), in this paper, we analytically deduce the evolution of the centre of instantaneous rotation (i.e., the fixed and mobile polodes) and the inflection circle for flexure hinges loaded by a concentrated force and moment, emphasizing the effect of the loading path.

This paper is organized as follows: In Section 2, a comprehensive deduction of the elasto-kinematics of flexure hinges and its analytical solution is discussed. In Section 3, the results of Section 2 are applied to deduce the analytical expressions for two fundamental instantaneous invariants: the fixed and mobile polodes and the inflection circle. Section 4 includes some numerical examples of the results obtained in Section 3 and experimental verification.

## 2. A Comprehensive Analytical Model of Flexure Hinge Kinematics

A faithful analytical characterization of rigid bodies connected via flexure hinges (Figure 1) should consider that, due to the high flexibility of the joints, the configuration changes involve large rotations/displacements of not only the rigid parts but also the deformable parts (although small strains are assumed) [53,54,55,56]. Therefore, it is necessary to use a fully nonlinear model.

**Figure 2:**
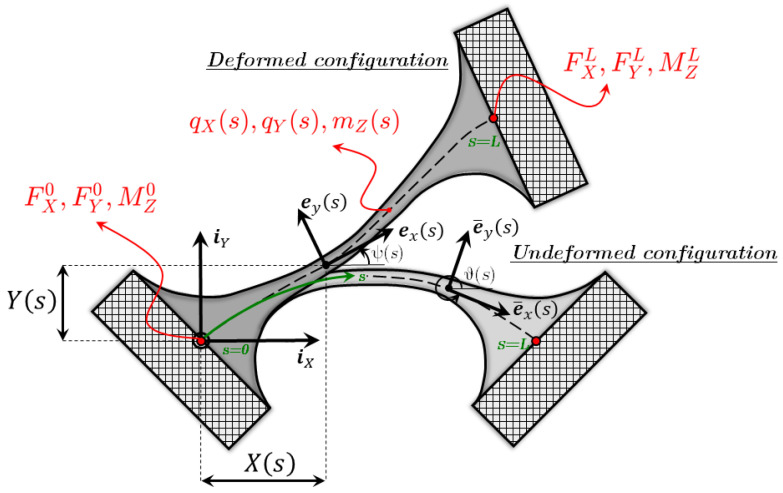
Generic undeformed and deformed configurations.

Figure 2 shows a generic 2D flexure hinge (curvilinear) in two positions. Three reference systems describe the deformed and undeformed configurations along the reference lines; these are parametrized by the curvilinear abscissa s∈[0,L], where L is the length of the undeformed configuration. The reference systems are: the global (inertial) frame, identified through the orthogonal unit vectors $i_{X},i_{Y}$ (for vector and tensor quantities, bold font is used), and two (local, non-inertial) mobile frames ${\bar{e}}_{x}\left(s\right),{\bar{e}}_{y}\left(s\right)$ and $e_{x}\left(s\right),e_{y}\left(s\right)$, the first associated with the undeformed configuration and the second with the deformed one. Given two-dimensional motion, the unit vector $i_{Z}=i_{X}\times i_{Y}$ is the same for all triads.

The two mobile frames can be expressed in Cartesian components (i.e., with respect to the global frame) through the change-of-basis orthogonal tensors $\Lambda^{\vartheta }$,$\;\Lambda^{\psi }$ as follows:

(1) $${\bar{e}}_{i}\left(s\right)=\Lambda_{\vartheta }\cdot i_{i}$$

(2) $$e_{i}\left(s\right)=\Lambda_{\psi }\cdot i_{i}$$

where the subscript i is used in place of X,Y or x,y.

(3) $$\begin{array}{l} \Lambda_{\vartheta }\left(s\right)={\bar{e}}_{x}⊗i_{X}+{\bar{e}}_{y}⊗i_{Y}\\ \;\;\;=\cos\vartheta \;i_{X}⊗i_{X}-\sin\vartheta \;i_{X}⊗i_{Y}+\sin\vartheta \;i_{Y}⊗i_{X}+\cos\vartheta \;i_{Y}⊗i_{Y} \end{array}$$

(4) $$\begin{array}{l} \Lambda_{\psi }\left(s\right)=e_{x}⊗i_{X}+e_{y}⊗i_{Y}\\ \;\;\;=\cos\psi \;i_{X}⊗i_{X}-\sin\psi \;i_{X}⊗i_{Y}+\sin\psi \;i_{Y}⊗i_{X}+\cos\psi \;i_{Y}⊗i_{Y} \end{array}$$

The angles ϑ(s),ψ(s) are shown in Figure 2.

Based on the previous equations, it is possible to define the curvatures of the reference lines, which are important intrinsic quantities that characterize the configurations. Differentiating Equations (1) and (2) with respect to s and using Equations (1) and (2) again to express the results in the mobile frame $e_{x}\left(s\right),e_{y}\left(s\right)$ results in the following:

(5) $$\frac{d{\bar{e}}_{i}\left(s\right)}{ds}=\bar{K}\cdot e_{i}=\bar{k}\times e_{i}$$

(6) $$\frac{de_{i}\left(s\right)}{ds}=K\cdot {\bar{e}}_{i}=k\times {\bar{e}}_{i}$$

in which:

(7) $$\bar{K}\left(s\right)=\frac{\partial \Lambda_{\vartheta }}{\partial s}{\left(\Lambda_{\vartheta }\right)}^{T}$$

(8) $$K\left(s\right)=\frac{\partial \Lambda_{\psi }}{\partial s}{\left(\Lambda_{\psi }\right)}^{T}$$

are the curvature tensors of the undeformed and deformed reference lines, respectively. The terms in Equations (7) and (8) are skew-symmetric tensors (Appendix A); therefore, it is possible to simplify Equations (5) and (6) using the curvature vectors, which are the axial vectors of the skew-symmetric curvature tensors:

(9) $$\bar{k}\left(s\right)=\frac{d\vartheta }{ds}\;i_{Z}$$

(10) $$k\left(s\right)=\frac{d\psi }{ds}\;i_{Z}$$

A one-dimensional model is adopted; therefore, for each point, the motion that occurs during the configuration change is due to two translation components along $e_{x},e_{y}$, and a rotation of the cross-section, assumed to be transversely rigid [57,58]. This allows us to separately examine axial (*ε*), shear (*γ*) and rotational (χ) strains. Adopting a Lagrangian approach, the radius vector that identifies the reference line of the deformed configuration is (Figure 2):

(11) $$r\left(s\right)=X\;i_{X}+Y\;i_{Y}$$

where X(s),Y(s) represent the position of the generic point of the deformed configuration with respect to the global reference system as functions of the curvilinear abscissa s.

The prime derivative of r is close to $e_{x}$, but the two vectors differ due to axial and shear strain:

(12) $$\frac{dr\left(s\right)}{ds}=\left(1+\varepsilon \right)e_{x}+\gamma \;e_{y}=\frac{\partial X}{\partial s}i_{X}+\frac{\partial Y}{\partial s}i_{Y}$$

Using the reverse of Equation (2), namely:

(13) $$i_{i}={\left(\Lambda_{\psi }\right)}^{T}\cdot e_{i}\left(s\right)$$

to express the right side of Equation (12) with respect to the mobile frame, the following relations occur:

(14) $$\varepsilon \left(s\right)=\frac{dX}{ds}\;\cos\psi +\frac{dY}{ds}\;\sin\psi -1$$

(15) $$\gamma \left(s\right)=-\frac{dX}{ds}\;\sin\psi +\frac{dY}{ds}\;\cos\psi$$

For slender structures, i.e., when the ratio between the half of thickness and the curvature radius is ≪1 [58,59], the rotational strain is:

(16) $$\chi \left(s\right)=\left(k-\bar{k}\right)\cdot i_{Z}=\frac{d\psi }{ds}-\frac{d\vartheta }{ds}$$

Therefore, the Green–Lagrange strains are given by:

(17) $$\varepsilon_{x}\left(s,\xi \right)=\varepsilon -y\chi$$

(18) $$\gamma_{xy}\left(s\right)=\gamma$$

where y is the coordinate along $e_{y}$ that identifies the points on the cross-section.

Assuming that the beam is made of an isotropic elastic material, the stress components are $\sigma_{x}=E\varepsilon_{x}\;,\;\tau_{xy}=G\gamma_{xy}$, where E,G are the axial end shear moduli of elasticity.

In this one-dimensional model, the flexure hinge exchanges forces and moments with the rigid bodies it connects through its ends. For convenience, the load quantities are referred to with respect to the global reference system. At s=0, the force and moment vectors are:

(19) $$F_{0}=F_{X}^{0}\;i_{X}+F_{Y}^{0}\;i_{Y}\;;M_{0}=M_{Z}^{0}\;i_{Z}$$

At the other end (s=L), the loads are:

(20) $$F_{L}=F_{X}^{L}\;i_{X}+F_{Y}^{L}\;i_{Y}\;;M_{L}=M_{Z}^{L}\;i_{Z}$$

Distributed forces and moments can be applied along the length of the flexure hinge:

(21) $$q\left(s\right)=q_{X}\left(s\right)\;i_{X}+q_{Y}\left(s\right)\;i_{Y}\;;m\left(s\right)=m_{Z}\left(s\right)\;i_{Z}$$

It is worth pointing out that distributed loads are usually omitted in studies regarding flexure hinges, as most of the forces are exchanged at the extremes. In this paper, we include this type of loads because there are some applications in which compliant mechanisms are driven using a distribution of smart materials (e.g., piezoelectric actuators [60] or shape memory alloys [61]); in these cases, the effect of smart-material actuators manifests as distributed loads.

At a generic point s, the forces and moment are given by:

(22) $$F\left(s\right)=F_{X}\left(s\right)i_{X}+F_{Y}\left(s\right)i_{Y}\;;M\left(s\right)=M_{Z}\left(s\right)i_{Z}$$

Since the flexure hinge respects the equilibrium in the deformed configuration, the forces and moment at the generic curvilinear abscissa s can be expressed as a function of the applied loads [62]. Assuming $F_{0},\;M_{0}$ at the origin, the following equations result:

(23) $$F_{X}\left(s\right)=-F_{X}^{0}-\overset{s}{\underset{0}{\int }}q_{X}\left(\tilde{s}\right)\;d\tilde{s}$$

(24) $$F_{Y}\left(s\right)=-F_{Y}^{0}-\overset{s}{\underset{0}{\int }}q_{Y}\left(\tilde{s}\right)\;d\tilde{s}$$

(25) $$M_{Z}\left(s\right)=-M_{Z}^{0}-YF_{X}^{0}+XF_{Y}^{0}+\overset{s}{\underset{0}{\int }}\left[\left(\tilde{Y}-Y\right)q_{X}\left(\tilde{s}\right)-\left(\tilde{X}-X\right)q_{Y}\left(\tilde{s}\right)-m_{Z}\left(\tilde{s}\right)\right]\;d\tilde{s}$$

where X(s),Y(s) depend on s, while $\tilde{X}\left(\tilde{s}\right),\tilde{Y}\left(\tilde{s}\right)$ depend on the dummy variable $\tilde{s}$.

Appendix B shows Equations (23)–(25), where known forces and moment $F_{L},M_{L}$ are given at s=L, and makes the relations between $F_{0},M_{0}$ and $F_{L},M_{L}$ explicit.

The axial and shear internal forces N(s),T(s) act in the normal and tangential direction of the cross-section; that is, rotated counterclockwise by a small angle γ with respect to the mobile frame $\;e_{x},e_{y}$. The effect of shear distortion on the direction of the internal forces is generally very small and can be neglected, thus obtaining:

(26) $$N\left(s\right)=\left(N-\gamma T\right)\;e_{x}+\left(T+\gamma N\right)\;e_{y}≅N\;e_{x}+T\;e_{y}$$

Therefore, the internal forces N(s) result from F(s), using Equation (2):

(27) $$N\left(s\right)=\Lambda_{\psi }\cdot F\left(s\right)$$

or, in components:

(28) $$N\left(s\right)=F_{X}\left(s\right)\cos\psi +F_{Y}\left(s\right)\sin\psi$$

(29) $$T\left(s\right)=-F_{X}\left(s\right)\sin\psi +F_{Y}\left(s\right)\cos\psi$$

Due to the planar motion, the internal moment is simply $M\left(s\right)=M_{z}\left(s\right)$.

The stress tensor can be expressed as follows (having neglected the shear distortion effect):

(30) $$\sigma \left(s,\xi \right)=\sigma_{x}\;e_{x}⊗e_{x}+\tau_{xy}\;\left(e_{x}⊗e_{y}+e_{y}⊗e_{x}\right)$$

The internal forces and moment N(s),M(s) can be expressed as the integration along the cross-section of the stress vector $t=\sigma \cdot e_{x}=\sigma_{x}\;e_{x}+\tau_{xy}\;e_{y}$:

(31) $$N\left(s\right)=\overset{}{\underset{A}{\int }}tdA\;\;\;\;;\;\;\;\;M\left(s\right)=\overset{}{\underset{A}{\int }}\left(y\;e_{y}\right)\times tdA$$

Using Equations (17) and (18) assuming the local reference as the principal of inertia and with the origin on the barycentre of the section, the force–strain relationships are:

(32) $$N\left(s\right)=EA\varepsilon \;\;\;;\;\;\;T\left(s\right)=GA_{s}\gamma \;\;\;;\;\;\;\;\;\;M\left(s\right)=EI\chi \;\;\;$$

where A(s),I(s) are the area and moment of the inertia of the cross-section and $A_{s}\left(s\right)$ is the effective shear area [57,63].

The three unknows that identify the deformed configuration are X,Y,ψ; they can be found by applying Equations (28), (29) and (32) to Equations (14)–(16):

(33) $$\frac{dX}{ds}=\cos\psi +\left(\frac{\cos^{2}\psi }{EA}+\frac{\sin^{2}\psi }{GA_{s}}\right)F_{X}\left(s\right)-\left(\frac{1}{GA_{s}}-\frac{1}{EA}\right)\sin\psi \cos\psi F_{Y}\left(s\right)\;$$

(34) $$\frac{dY}{ds}=\sin\psi -\left(\frac{1}{GA_{s}}-\frac{1}{EA}\right)\sin\psi \cos\psi F_{X}\left(s\right)+\left(\frac{\sin^{2}\psi }{EA}+\frac{\cos^{2}\psi }{GA_{s}}\right)F_{Y}\left(s\right)$$

(35) $$\frac{d\psi }{ds}=\frac{d\vartheta }{ds}+\frac{M\left(s\right)}{EI}$$

Equations (33)–(35) form a nonlinear first-order ODE system that holds for every type of flexure hinge (with variable section, initially curvilinear, etc.). It is not possible to solve this analytically in a general form (i.e., for all types of load conditions) [37,39,54]. The boundary conditions (b.c.) in Equations (33) and (34) are trivial, i.e., $X\left(s=0\right)=X_{0}\;,Y\left(s=0\right)=Y_{0}$, namely the choice of the location of the global reference system. More interesting are the b.c. of the Equation (35), which represent the difficulties encountered in solving this system. In general, $\psi \left(s=0\right)=\psi_{0}$ is unknown; however, above all, the bending moment $M_{Z}^{0}$ at the origin (namely, the curvature $\psi^{\prime }\left(s=0\right)=\psi_{0}^{\prime }$) is unknown. The b.c. regarding the bending moment could be known at the end s=L (e.g., the case of a cantilever beam loaded by concentrated forces at the end), which entails that the b.c. problem becomes a boundary value problem (b.v.p.). As is well known, the numerical methods employed to solve ODE only work with initial value problems (i.v.p.); therefore, to solve a b.v.p., a shooting method should be adopted [37,39] that involves integrating the systems of Equations (33)–(35) several times.

Often, Equation (35) appears as a second-order ODE. By applying the derivative of Equation (35), being careful to use the Leibniz integration rule (differentiation under the integral sign) for the differentiation of Equation (25), one obtains:

(36) $$\frac{d^{2}\psi }{ds^{2}}+\left(\frac{1}{EI}\frac{d^{2}EI}{ds^{2}}\right)\;\frac{d\psi }{ds}=\frac{d^{2}\vartheta }{ds^{2}}+\frac{1}{EI}\left[\left(\frac{d\;EI}{ds}\right)\frac{d\vartheta }{ds}+\frac{dy}{ds}F_{X}\left(s\right)-\frac{dx}{ds}F_{Y}\left(s\right)+m_{z}\right]$$

This form does not change the aforementioned difficulties. $\psi_{0}^{\prime }$ and $\psi_{0}$ remain unknown; however, the form of Equation (36) can be analytically integrated under some assumptions. This will be carried out in the following section to provide some benchmark results regarding the computation of the fixed and mobile polodes of compliant mechanisms.

**Figure 3:**
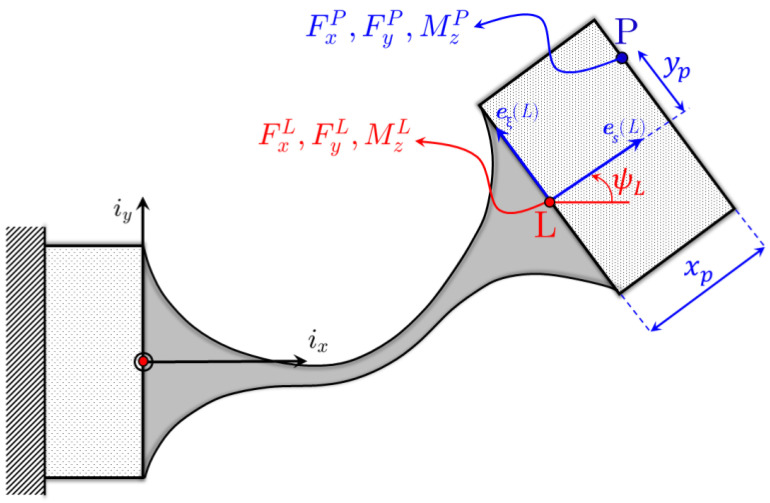
Flexure hinge loaded by forces and moment applied to a generic point of the rigid body connected to it.

Furthermore, it is important to emphasize (for what follows) that in compliant mechanism applications, the forces and moments are not directly applied at the ends of the flexure hinges but on the rigid bodies connected with them. In this case, the forces and moments $F_{X}^{0},F_{Y}^{0},M_{Z}^{0}$ or $F_{X}^{L},F_{Y}^{L},M_{Z}^{L}$ applied to the flexure hinge are also a function of the unknown angles $\psi_{0}$ or $\psi_{L}$ (Figure 3). Consider Figure 3, where a flexure hinge connects two rigid bodies, of which the one on the left is clamped. The rigid body on the right is loaded at point P with the forces and moment:

(37) $$F_{P}=F_{X}^{P}\;i_{X}+F_{Y}^{P}\;i_{Y};M_{P}=M_{Z}^{P}\;i_{Z}$$

Applying static equivalence, the forces and moment $F_{X}^{L},F_{Y}^{L},M_{Z}^{L}$ experienced by the flexure hinge are not only a function of known quantities such as $F_{X}^{P},F_{Y}^{P},M_{Z}^{P}$ and $x_{P},y_{P}$, but also of the unknown angle $\psi_{L}$ (or $\psi_{0}$):

(38) $$F_{X}^{L}=F_{X}^{P}$$

(39) $$F_{Y}^{L}=F_{Y}^{P}$$

(40) $$\begin{array}{l} M_{Z}^{L}=M_{Z}^{P}+\left[x_{P}\;e_{x}\left(L\right)+y_{P}\;e_{y}\left(L\right)\right]\times \left(F_{X}^{P}\;\;i_{X}+F_{Y}^{P}\;\;i_{Y}\right)\\ \;\;\;=M_{Z}^{P}+\left(x_{P}\cos\psi_{L}-y_{P}\sin\psi_{L}\right)\;F_{Y}^{P}-\left(x_{P}\sin\psi_{L}-y_{P}\cos\psi_{L}\right)\;F_{X}^{P} \end{array}$$

Therefore, as previously mentioned, in this scenario the applied moment $M_{Z}^{L}$ depends on the unknown angle $\psi_{L}$.

This case is an example in which the b.c. turn into a b.v.p., inasmuch the moment $M_{Z}^{0}$ at the origin is unknown; however, it must be found such that at the end of the computation, the final moment $M_{Z}^{L}$ obtained from the curvature $\psi^{\prime }\left(L\right)$ is consistent with Equation (40). In the following section, an analytical solution of Equations (33)–(35) is presented under some simplifying assumptions.

### 2.1. Analytical Solution

An analytical solution of Equations (33), (34) and (36) can be found taking into account some assumptions: the extensional and shear strains are negligible $\left(\varepsilon =\gamma =0\;or\;EA,GA_{s}\to \infty \right)$; the section has a constant shape (EI=const.); the initial curvature is constant $\left(\vartheta^{\prime }=const.\right)$; and the distributed loads are null $\left(q_{X}=q_{Y}=m_{Z}=0\right)$. Although the analytical solution requires the assumption of a constant section, this is a valuable solution since for notched flexure hinges, the main deformable parts are the central ones with an extended constant section (Figure 3).

Under these conditions, the forces $F_{X}\;,\;F_{Y}$ (Equations (23) and (24) or Equations (A3) and (A4)) acting at a generic point s are constant, and the Equations (33), (34) and (36) can be simplified as:

(41) $$\frac{dX}{ds}=\cos\psi$$

(42) $$\frac{dY}{ds}=\sin\psi$$

(43) $$EI\;\frac{d^{2}\psi }{ds^{2}}=F_{X}\sin\psi -F_{Y}\cos\psi$$

Multiplying both sides of the latter equation by $\psi^{\prime }$, Equation (43) can be integrated, obtaining:

(44) $$\frac{EI}{2}{\left(\frac{d\psi }{ds}\right)}^{2}=c-F_{X}\cos\psi -F_{Y}\sin\psi$$

where c is an integration constant.

If b.c. at s=0 are applied:

(45) $$c=F_{X}\cos\psi_{0}+F_{Y}\sin\psi_{0}+\frac{EI}{2}{\left(\vartheta^{\prime }+\frac{M_{Z}^{0}}{EI}\right)}^{2}$$

Otherwise, if b.c. at s=L are applied:

(46) $$c=F_{X}\cos\psi_{L}+F_{Y}\sin\psi_{L}+\frac{EI}{2}{\left(\vartheta^{\prime }+\frac{M_{Z}^{L}}{EI}\right)}^{2}$$

Equation (44) can be rearranged as follows:

(47) $$\frac{d\psi }{ds}=sign\left(\psi^{\prime }\right)\;f\left(\psi \right)$$

in which:

(48) $$f\left(\psi \right)=\sqrt{\frac{2}{EI}\left(c-F_{X}\cos\psi -F_{Y}\sin\psi \right)}$$

The function $sign\left(\psi^{\prime }\right)$ is unknown and generally piecewise-defined; it defines the sign of the curvature. This is a crucial point; since the ODE system in Equations (41)–(43) is nonlinear, more than one solution generally exists. These multiple solutions of the deformed shape have an unknown number of inflection points (i.e., points where the curvature $\psi^{\prime }=0$ and the curvature sign therefore changes). Furthermore, the presence of one or more inflection points depends on the position of the applied load in the deformed (unknown) configuration. A priori determination of the presence of inflection points (i.e., the exact determination of $sign\left(\psi^{\prime }\right)$) as the only function of the magnitude of the applied loads is an open problem. We will not deal with that in the following and present a solution limited to one internal inflection point.

If no internal inflection points exist, the angle ψ(s) is monotone and the sign function is trivial:

(49) $$sign\left(\psi^{\prime }\right)=\pm 1\;\;\;\;\forall \;\;\;\psi \left(s\right)\in \left(\psi_{0},\psi_{L}\right)$$

However, the latter can be zero at the extremities if the terms $\left(\vartheta^{\prime }+\frac{M_{Z}^{0}}{EI}\right)$ or $\left(\vartheta^{\prime }+\frac{M_{Z}^{L}}{EI}\right)$ are nulls in $\psi_{0}$ or $\psi_{L}$.

If one internal inflection point does exist, Equation (47) is null at a point $s=s_{in}$, which corresponds to an angle $\psi \left(s=s_{in}\right)=\psi_{in}$:

(50) $$F_{X}\cos\psi_{in}+F_{Y}\sin\psi_{in}=c$$

The latter equations can be manipulated to obtain a relation between the angle $\psi_{in}$ and the triplet $\psi_{0},\vartheta^{\prime },M_{Z}^{0}$ or $\psi_{L},\vartheta^{\prime },M_{Z}^{L}$, according to the choice of the Equation (45) or Equation (46) for c evaluation:

(51) $$\psi_{in}=\arcsin\left(\frac{c}{\sqrt{F_{X}{}^{2}+F_{Y}{}^{2}}}\right)-\varphi$$

where:

(52) $$\varphi =\mathrm{atan}2\left(F_{Y},F_{X}\right)$$

We suspect that if multiple inflection points $\psi_{in,1}\;,\psi_{in,2},\;\ldots ,\;\psi_{in,k}$ exist, the relation between the generic inflection point angle $\psi_{in,k}$ and the angle $\psi_{0}$ or $\psi_{L}$ can always be found with Equation (50); however, the sign equation and the closure equation (Equation (54)) must be split into parts. This issue has not yet been thoroughly investigated and is beyond the scope of the present paper.

The sign function for a single inflection point appears in a more articulated form than Equation (49), namely as a piecewise-defined function:

(53) $$sign\left(\psi^{\prime }\right)=\{\begin{array}{c} sign\left(\psi_{0}^{\prime }\right)\;\;\;\;if\;\;\;\psi \left(s\right)\in \left(\psi_{0},\psi_{in}\right)\\ 0\;\;\;\;\;\;\;\;\;\;\;\;\;if\;\;\;\;\;\psi \left(s\right)=\psi_{in}\;\\ sign\left(\psi_{L}^{\prime }\right)\;\;\;\;if\;\;\;\psi \left(s\right)\in \left(\psi_{in},\psi_{L}\right) \end{array}$$

where $sign\left(\psi_{0}^{\prime }\right)\;,\;sign\left(\psi_{L}^{\prime }\right)$ are constant values that can be ±1. Again, Equation (53) can be zero at the extremities if the terms $\left(\vartheta^{\prime }+\frac{M_{Z}^{0}}{EI}\right)$ or $\left(\vartheta^{\prime }+\frac{M_{Z}^{L}}{EI}\right)$ are nulls in $\psi_{0}$ or $\psi_{L}$.

Both in the case of zero or a single inflection point, the determination of $\psi_{in}$ is conditioned by the knowledge of $\psi_{0}$ or $\psi_{L}$. To find the unknown angle, it is necessary to integrate Equation (47), obtaining:

(54) $$L=\overset{\psi_{L}}{\underset{\psi_{0}}{\int }}\frac{sign\left(\psi^{\prime }\right)\;d\psi }{f\left(\psi \right)}$$

Equation (54) appears as a closure equation that involves geometric and material variables, in addition to applied loads. It is not possible to analytically integrate Equation (54), and the search for the unknown parameter $\left(\psi_{0}\;or\;\psi_{L}\right)$ involves an attempt method [53,54]. It is important to observe that the function f(ψ) also depends on $\psi_{L}$.

Once Equation (54) is solved with the considered geometry and loads, the deformed shape can be obtained through the integration of Equations (41) and (42), using the relation $ds=d\psi /\psi^{\prime }$ and applying Equation (47) and the b.c. $X_{0}=X\left(s=0\right)\;,Y_{0}=Y\left(s=0\right)$:

(55) $$X\left(\psi \right)=X_{0}+\overset{\psi }{\underset{\psi_{0}}{\int }}\;\frac{sign\left({\tilde{\psi }}^{\prime }\right)\cos\tilde{\psi }\;}{f\left(\tilde{\psi }\right)}d\tilde{\psi }$$

(56) $$Y\left(\psi \right)=Y_{0}+\int_{\psi_{0}}^{\psi }\;\frac{sign\left({\tilde{\psi }}^{\prime }\right)\cos\tilde{\psi }\;}{f\left(\tilde{\psi }\right)}d\tilde{\psi }$$

where $\tilde{\psi }$ is a dummy variable and $\psi \left(s\right)\in \left[\psi_{0},\psi_{L}\right]$.

Similar to Equation (54), it is not possible to analytically integrate Equations (55) and (56); thus, they require a numerical integration. However, Equations (54)–(56) are computationally advantageous when compared to the full-length numerical integration required to compute Equations (33)–(35); this is because Equations (54)–(56) allow for the computation of the results only at a single point (e.g., the end point), which is very advantageous in the computation of the instantaneous invariants covered in the following section.

If an inflection point exists, Equations (54)–(56) involve improper integrals. To avoid complications due to singularity, Equations (54)–(56) are evaluated by applying a trick reported in Appendix C.

## 3. Analytical Deduction of Instantaneous Invariants for Compliant Mechanism

The kinematic synthesis of rigid planar mechanisms is often performed using instantaneous geometric and kinematic invariants [64,65,66,67,68,69]. The first type of invariants (geometric) are more useful, as they have the important property of being independent of the motion time law. They include important geometric loci, such as the fixed and mobile polodes (and their curvature, appearing in the Euler–Savary formula), the first Bresse’s circle (zero normal acceleration), the cubic curve of stationary curvature, Ball’s point and the Burmester points. The second type of invariants (kinematic) define instantaneous properties of the motion but are a function of the motion time law (i.e., angular velocity, acceleration, etc.). Some examples of instantaneous kinematic invariants are the second Bresse’s circle (zero tangential acceleration), the centre of the accelerations (i.e., the point with null acceleration), the jerk and Javot centres, etc.

Instantaneous invariants, mainly the geometric ones, are essential to set problems of kinematic synthesis in analytical form [68,69,70,71,72,73].

To our best knowledge, for compliant mechanisms, the instantaneous invariants have not yet been deduced in an analytical form. As mentioned in Section 1, pseudo-rigid models are commonly used [2,44,45], in which the flexure hinges are replaced by rigid bars connected with lumped hinges. However, this approach implies that the bar lengths and the positions of the lumped hinges must be changed during motion as the centre of instantaneous rotation moves, and their positions change as a function of the applied load.

In this section, the determination of the instantaneous invariants is addressed with a direct approach, considering the real deformable behaviour of flexure hinges.

**Figure 4:**
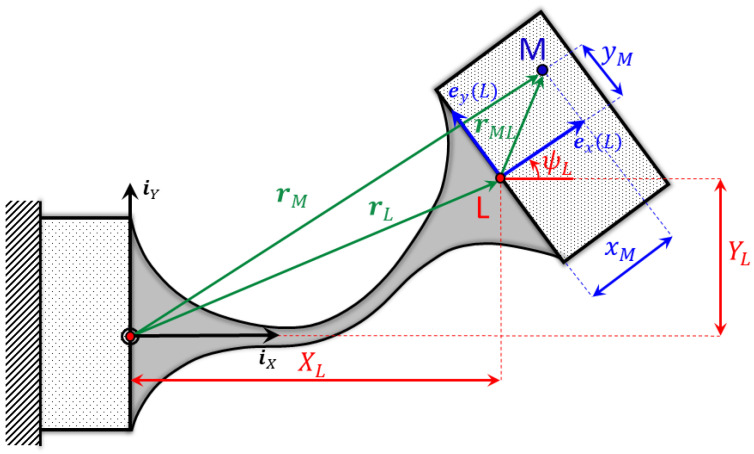
Generic configuration of two rigid bodies (fixed and mobile) connected by a flexure hinge.

The first instantaneous invariant investigated is the centre of instantaneous rotation. In order to study the relative motion, the case of a flexure hinge connected a fixed and a mobile rigid body is taken into account. The position of a generic point M on the mobile rigid body in Figure 4 is:

(57) $$r_{M}=r_{L}+r_{ML}$$

where:

(58) $$r_{M}=X_{M}\;i_{X}+Y_{M}\;i_{Y}$$

(59) $$r_{L}=X_{L}\;i_{X}+Y_{L}\;i_{Y}\;\;\;$$

(60) $$r_{ML}=x_{M}\;e_{x}\left(L\right)+y_{M}\;e_{y}\left(L\right)=\Lambda_{\psi_{L}}\cdot x_{ML}$$

in which Equation (2) has been used in Equation (60). The others terms that appear in Equations (59) and (60) are: $X_{L}=X\left(\psi =\psi_{L}\right)\;,\;Y_{L}=Y\left(\psi =\psi_{L}\right)$, $x_{ML}=x_{M}\;i_{X}+y_{M}\;i_{Y}$ and $\Lambda_{\psi_{L}}=\Lambda_{\psi }\left(\psi =\psi_{L}\right)$.

In other words, $X_{M},Y_{M}$ are the coordinates of the generic point M with respect to the global reference system, while $x_{M},y_{M}$ are the coordinates of the same point with respect to the mobile frame $e_{x}\left(\psi_{L}\right),e_{y}\left(\psi_{L}\right)$, having its origin at the end of the flexure hinge.

The coordinates of the centre of instantaneous rotation (c.i.r.) ($X_{C},Y_{C}$, still unknown) of the mobile rigid body expressed in the global reference system do not change for an infinitesimal motion:

(61) $$dr_{C}=0=dr_{L}+dr_{CL}$$

The coordinates $x_{C},y_{C}$ of the c.i.r., expressed with respect to the mobile frame are not modified during infinitesimal motion due to the rigidity of the mobile rigid body. The only variable terms are $X_{L},Y_{L}$ and $e_{x}\left(\psi_{L}\right),e_{y}\left(\psi_{L}\right)$, which are all functions of the final angle $\psi_{L}$ (Equations (2), (55) and (56)). Hence:

(62) $$\frac{dr_{C}}{d\psi_{L}}=0=\frac{dr_{L}}{d\psi_{L}}+\frac{d\Lambda_{\psi_{L}}}{d\psi_{L}}\cdot x_{CL}\underset{}{\Rightarrow }x_{CL}=-{\left(\frac{d\Lambda_{\psi_{L}}}{d\psi_{L}}\right)}^{T}\frac{dr_{L}}{d\psi_{L}}$$

Or, in components:

(63) $$x_{C}\left(\psi_{L}\right)=\frac{dX_{L}}{d\psi_{L}}\;\sin\psi_{L}-\frac{dY_{L}}{d\psi_{L}}\;\cos\psi_{L}$$

(64) $$y_{C}\left(\psi_{L}\right)=\frac{dX_{L}}{d\psi_{L}}\;\cos\psi_{L}+\frac{dY_{L}}{d\psi_{L}}\;\sin\psi_{L}$$

Equations (63) and (64) are the Cartesian parametric equations of the mobile polode, namely the position of the c.i.r. within the mobile frame. Using Equations (57), (60) and (62), the equation of the fixed polode is given as:

(65) $$r_{C}=r_{L}-\Lambda_{\psi_{L}}\cdot {\left(\frac{d\Lambda_{\psi_{L}}}{d\psi_{L}}\right)}^{T}\frac{dr_{L}}{d\psi_{L}}$$

From Equation (57), the condition of stationarity yields:

(66) $$X_{C}\left(\psi_{L}\right)=X_{L}-\frac{dY_{L}}{d\psi_{L}}\;$$

(67) $$Y_{C}\left(\psi_{L}\right)=Y_{L}+\frac{dX_{L}}{d\psi_{L}}\;$$

Equations (63), (64), (66) and (67) are generally valid no matter the shape of the flexure hinges. In the following, they are made explicit by taking into account the case examined in Section 2.1, in which the flexure hinge is loaded by different combinations of loads $F_{X}^{P},F_{Y}^{P},M_{Z}^{P}$; this scenario can be analytically explained using Equations (55) and (56). Although Equations (55) and (56) are valid for flexure hinges with a constant section only, all results obtained through the use of Equations (55) and (56) may be extended to notched flexure hinges if an equivalent length of the main deformable part (with a constant section) is estimated [73].

By computing Equations (55) and (56) with $\psi =\psi_{L}$ and applying their differentiation with respect to $\psi_{L}$, considering that the terms c, $F_{X}$, $F_{Y}$ are function of $\psi_{L}$, the following result is obtained:

(68) $$\frac{dX_{L}}{d\psi_{L}}=\frac{sign\left(\psi_{L}^{\prime }\right)\cos\psi_{L}\;}{\left(\vartheta^{\prime }+\frac{M_{Z}^{L}}{EI}\right)}-\overset{\psi_{L}}{\underset{\psi_{0}}{\int }}sign\left(\psi^{\prime }\right)\frac{df\left(\psi \right)}{d\psi_{L}}\;\frac{\cos\psi }{\;f{\left(\psi \right)}^{2}}d\psi$$

(69) $$\frac{dY_{L}}{d\psi_{L}}=\frac{sign\left(\psi_{L}^{\prime }\right)\sin\psi_{L}\;}{\left(\vartheta^{\prime }+\frac{M_{Z}^{L}}{EI}\right)}-\overset{\psi_{L}}{\underset{\psi_{0}}{\int }}sign\left(\psi^{\prime }\right)\frac{df\left(\psi \right)}{d\psi_{L}}\;\frac{\sin\psi }{\;f{\left(\psi \right)}^{2}}d\psi$$

where, using Equations (38), (39), (A3) and (A4) for the derivatives of $F_{X},F_{Y}$:

(70) $$\frac{df\left(\psi \right)}{d\psi_{L}}=\frac{1}{EI\;f\left(\psi \right)}\left[\frac{dc}{d\psi_{L}}-\frac{dF_{X}^{P}}{d\psi_{L}}\cos\psi -\frac{dF_{Y}^{P}}{d\psi_{L}}\sin\psi \right]$$

Differentiating Equation (46) gives the following:

(71) $$\frac{dc}{d\psi_{L}}=\frac{dF_{X}^{P}}{d\psi_{L}}\cos\psi_{L}-F_{X}^{P}\sin\psi_{L}+\frac{dF_{Y}^{P}}{d\psi_{L}}\sin\psi_{L}+F_{Y}^{P}\cos\psi_{L}+\left(\vartheta^{\prime }+\frac{M_{Z}^{L}}{EI}\right)\;\frac{dM_{Z}^{L}}{d\psi_{L}}$$

in which $M_{Z}^{L}$ is reported in Equation (40), and its derivative is:

(72) $$\begin{array}{c} \frac{dM_{Z}^{L}}{d\psi_{L}}=\frac{dM_{Z}^{P}}{d\psi_{L}}+\left[\left(\frac{dF_{Y}^{P}}{d\psi_{L}}-F_{X}^{P}\right)x_{P}+\left(\frac{dF_{X}^{P}}{d\psi_{L}}-F_{Y}^{P}\right)y_{P}\right]\cos\psi_{L}+\\ -\left[\left(\frac{dF_{X}^{P}}{d\psi_{L}}+F_{Y}^{P}\right)x_{P}+\left(\frac{dF_{Y}^{P}}{d\psi_{L}}+F_{X}^{P}\right)y_{P}\right]\sin\psi_{L} \end{array}$$

**Figure 5:**
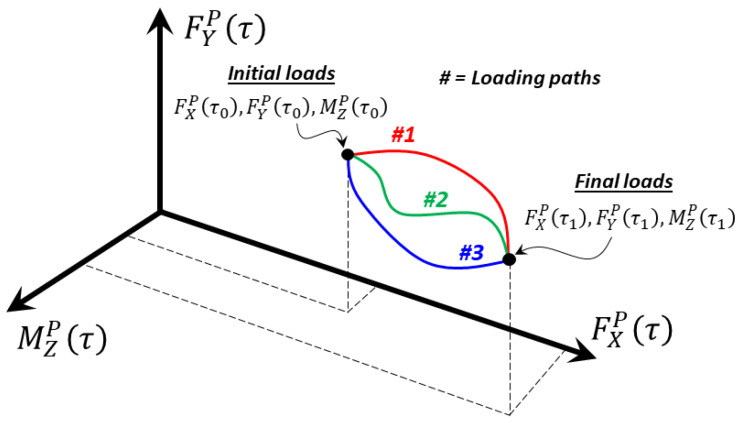
Generic loading paths.

A dimensionless parameter $\tau \in \left[\tau_{0},\tau_{1}\right]$ can be introduced to “chronologically” evaluate the trend of the loading path. In other words, the parameter τ acts as an ordering variable, identifying the configuration change as a function of it. Therefore, the applied loads become a function of τ $F_{X}^{P}\left(\tau \right),F_{X}^{P}\left(\tau \right),F_{X}^{P}\left(\tau \right)$, where the loads applied at the initial and final configurations are $F_{X}^{P}\left(\tau_{0}\right),F_{Y}^{P}\left(\tau_{0}\right),M_{Z}^{P}\left(\tau_{0}\right)$ and $F_{X}^{P}\left(\tau_{1}\right),F_{Y}^{P}\left(\tau_{1}\right),M_{Z}^{P}\left(\tau_{1}\right)$, respectively (Figure 5).

As a consequence, the final angle $\psi_{L}\left(\tau \right)$ becomes a function of the parameter τ, and the differentiations that appear in Equations (70)–(72) can be expressed as:

(73) $$\frac{dF_{X}^{P}}{d\psi_{L}}=\frac{\dot{F_{X}^{P}}}{\dot{\psi_{L}}};\frac{dF_{Y}^{P}}{d\psi_{L}}=\frac{\dot{F_{Y}^{P}}}{\dot{\psi_{L}}};\frac{dM_{Z}^{P}}{d\psi_{L}}=\frac{\dot{M_{Z}^{P}}}{\dot{\psi_{L}}}$$

where the notation $\dot{\left(\right)}$ indicates the derivatives with respect to the parameter τ.

To recap, for one d.o.f. rigid mechanisms, $\psi_{L}$ is a function of time t and the relationship is unique. This implies that the polodes are instantaneous geometric invariants. For a compliant mechanism, $\psi_{L}$ is not only a function of the applied loads’ intensity but also of the loading histories and loading rates. In other words, the polodes are not conservative; if two different loading paths are applied (e.g., two different motion time laws to obtain two different dynamic loads), the c.i.r. locations (i.e., fixed and mobile polodes) differ. Therefore, the polodes are not instantaneous geometric invariants; as consequence, for compliant mechanisms do not exist instantaneous invariants that are independent of the motion time law.

There is only one situation where, for static loading, the polodes are conservative (i.e., are not loading-path dependent); this occurs when the flexure hinges are loaded by a concentrated moment only. For this case, the analytical equations of the fixed and mobile polodes are provided in Appendix D.

Another important instantaneous invariant that is worth defining analytically is the first Bresse’s circle (or inflection circle) [68,69,70,71,72,74,75]. It is the locus of points that have an instantaneous rectilinear motion (i.e., have zero normal acceleration). The curvature of the trajectory of a generic moving point M of the mobile rigid body (Figure 4) is:

(74) $$k_{M}=\frac{\frac{dX_{M}}{d\psi_{L}}\;\frac{d^{2}Y_{M}}{d\psi_{L}{}^{2}}-\frac{d^{2}X_{M}}{d\psi_{L}{}^{2}}\;\frac{dY_{M}}{d\psi_{L}}}{{\left[{\left(\frac{dX_{M}}{d\psi_{L}}\right)}^{2}+{\left(\frac{dY_{M}}{d\psi_{L}}\right)}^{2}\right]}^{\frac{3}{2}}}\;$$

To find the locus of points $X_{in},Y_{in}$, which have zero normal acceleration (i.e., an instantaneous inflection in their trajectory) and hence zero curvature to their trajectory, it is sufficient to set Equation (74) to zero:

(75) $$\frac{dX_{in}}{d\psi_{L}}\;\frac{d^{2}Y_{in}}{d\psi_{L}{}^{2}}-\frac{d^{2}X_{in}}{d\psi_{L}{}^{2}}\;\frac{dY_{in}}{d\psi_{L}}=0$$

Using Equation (57), one obtains:

(76) $$\frac{dX_{in}}{d\psi_{L}}=\frac{dX_{L}}{d\psi_{L}}-x_{in}\;\sin\psi_{L}-y_{in}\;\cos\psi_{L}$$

(77) $$\frac{d^{2}X_{in}}{d\psi_{L}{}^{2}}=\frac{d^{2}X_{L}}{d\psi_{L}{}^{2}}-x_{in}\;\cos\psi_{L}+y_{in}\;\sin\psi_{L}$$

(78) $$\frac{dY_{in}}{d\psi_{L}}=\frac{dY_{L}}{d\psi_{L}}+x_{in}\;\cos\psi_{L}-y_{in}\;\sin\psi_{L}$$

(79) $$\frac{d^{2}Y_{in}}{d\psi_{L}{}^{2}}=\frac{d^{2}Y_{L}}{d\psi_{L}{}^{2}}-x_{in}\;\sin\psi_{L}-y_{in}\;\cos\psi_{L}$$

Applying Equations (76)–(79), Equation (75) is expressed as:

(80) $$x_{in}{}^{2}+y_{in}{}^{2}+a\;x_{in}+b\;y_{in}+c=0$$

where:

(81) $$a=\left(\frac{dY_{L}}{d\psi_{L}}-\frac{d^{2}X_{L}}{d\psi_{L}{}^{2}}\right)\cos\psi_{L}-\left(\frac{dX_{L}}{d\psi_{L}}+\frac{d^{2}Y_{L}}{d\psi_{L}{}^{2}}\right)\sin\psi_{L}$$

(82) $$b=\left(\frac{d^{2}X_{L}}{d\psi_{L}{}^{2}}-\frac{dY_{L}}{d\psi_{L}}\right)\sin\psi_{L}-\left(\frac{d^{2}Y_{L}}{d\psi_{L}{}^{2}}+\frac{dX_{L}}{d\psi_{L}}\right)\cos\psi_{L}$$

(83) $$c=\frac{dX_{L}}{d\psi_{L}}\;\frac{d^{2}Y_{L}}{d\psi_{L}{}^{2}}-\frac{d^{2}X_{L}}{d\psi_{L}{}^{2}}\;\frac{dY_{L}}{d\psi_{L}}$$

Equation (80) is a circumference. Therefore, the parametric equations of the inflection circle with respect to the mobile frame are:

(84) $$x_{in}=c_{x}+R\cos u$$

(85) $$y_{in}=c_{y}+R\sin u\;$$

where u∈[0,2π] is the curve parameter and $C=c_{x}\;i_{X}+c_{y}\;i_{Y}=-\frac{1}{2}\left[a\;i_{X}+b\;i_{Y}\right]$ and $R=\frac{1}{2}\sqrt{a^{2}+b^{2}-4c}$ are the centre and radius of the inflection circle in Equation (84,85), respectively. The parametric Cartesian equations of the inflection circle with respect the fixed frame are (Equation (57)):

(86) $$X_{in}=X_{L}+x_{in}\;\cos\psi_{L}-y_{in}\;\sin\psi_{L}$$

(87) $$Y_{in}=Y_{L}+x_{in}\;\sin\psi_{L}+y_{in}\;\cos\psi_{L}\;$$

Equations (84)–(87) of the inflection circle are analytically defined if the second derivative of $X_{L},Y_{L}$ is made explicit (the prime derivatives are already defined by Equations (68) and (69)).

Therefore, by differentiating Equations (68) and (69), one obtains:

(88) $$\begin{array}{c} \frac{d^{2}X_{L}}{d\psi_{L}{}^{2}}=\frac{sign\left(\psi_{L}^{\prime }\right)\;\cos\psi_{L}\;}{EI{\left(\vartheta^{\prime }+\frac{M_{Z}^{L}}{EI}\right)}^{3}}\left[F_{Y}^{P}\cos\psi_{L}-F_{X}^{P}\sin\psi_{L}+\left(\vartheta^{\prime }+\frac{M_{Z}^{L}}{EI}\right)\frac{dM_{Z}^{L}}{d\psi_{L}}\right]\\ -\frac{sign\left(\psi_{L}^{\prime }\right)\sin\psi_{L}\;}{\left(\vartheta^{\prime }+\frac{M_{Z}^{L}}{EI}\right)}-\frac{sign\left(\psi_{L}^{\prime }\right)\cos\psi_{L}\;}{EI{\left(\vartheta^{\prime }+\frac{M_{Z}^{L}}{EI}\right)}^{2}}\;\frac{dM_{Z}^{L}}{d\psi_{L}}\\ -\overset{\psi_{L}}{\underset{\psi_{0}}{\int }}\frac{sign\left(\psi^{\prime }\right)\;\cos\psi }{f{\left(\psi \right)}^{2}}\left[\frac{d^{2}f\left(\psi \right)}{d\psi_{L}{}^{2}}-\frac{2}{f\left(\psi \right)}{\left(\frac{df\left(\psi \right)}{d\psi_{L}}\right)}^{2}\right]d\psi \end{array}$$

(89) $$\begin{array}{c} \frac{d^{2}Y_{L}}{d\psi_{L}{}^{2}}=\frac{sign\left(\psi_{L}^{\prime }\right)\;\sin\psi_{L}\;}{EI{\left(\vartheta^{\prime }+\frac{M_{Z}^{L}}{EI}\right)}^{3}}\left[F_{Y}^{P}\cos\psi_{L}-F_{X}^{P}\sin\psi_{L}+\left(\vartheta^{\prime }+\frac{M_{Z}^{L}}{EI}\right)\frac{dM_{Z}^{L}}{d\psi_{L}}\right]\\ -\frac{sign\left(\psi_{L}^{\prime }\right)\cos\psi_{L}\;}{\left(\vartheta^{\prime }+\frac{M_{Z}^{L}}{EI}\right)}-\frac{sign\left(\psi_{L}^{\prime }\right)\sin\psi_{L}\;}{EI{\left(\vartheta^{\prime }+\frac{M_{Z}^{L}}{EI}\right)}^{2}}\;\frac{dM_{Z}^{L}}{d\psi_{L}}\\ -\overset{\psi_{L}}{\underset{\psi_{0}}{\int }}\frac{sign\left(\psi^{\prime }\right)\;\sin\psi }{f{\left(\psi \right)}^{2}}\left[\frac{d^{2}f\left(\psi \right)}{d\psi_{L}{}^{2}}-\frac{2}{f\left(\psi \right)}{\left(\frac{df\left(\psi \right)}{d\psi_{L}}\right)}^{2}\right]d\psi \end{array}$$

where:

(90) $$\begin{array}{c} \frac{d^{2}f\left(\psi \right)}{d\psi_{L}{}^{2}}=\frac{1}{EI\;f\left(\psi \right)}\left[\frac{d^{2}c}{d\psi_{L}{}^{2}}+\left(\frac{dF_{Y}^{P}}{d\psi_{L}}-\frac{d^{2}F_{X}^{P}}{d\psi_{L}{}^{2}}\right)\cos\psi +\left(\frac{dF_{X}^{P}}{d\psi_{L}}-\frac{d^{2}F_{Y}^{P}}{d\psi_{L}{}^{2}}\right)\sin\psi \right]\\ +\frac{1}{f\left(\psi \right)}\;{\left(\frac{df\left(\psi \right)}{d\psi_{L}}\right)}^{2} \end{array}$$

(91) $$\begin{array}{c} \frac{d^{2}c}{d\psi_{L}{}^{2}}=\left(\frac{d^{2}F_{X}^{P}}{d\psi_{L}{}^{2}}-F_{X}^{P}+\frac{dF_{Y}^{P}}{d\psi_{L}}\right)\cos\psi_{L}+\left(\frac{d^{2}F_{Y}^{P}}{d\psi_{L}{}^{2}}-\frac{dF_{X}^{P}}{d\psi_{L}}-F_{Y}^{P}\right)\sin\psi_{L}\\ +{\left(\frac{1}{EI}\frac{dM_{Z}^{L}}{d\psi_{L}}\right)}^{2}+\left(\vartheta^{\prime }+\frac{M_{Z}^{L}}{EI}\right)\;\frac{d^{2}M_{Z}^{L}}{d\psi_{L}{}^{2}} \end{array}$$

(92) $$\begin{array}{c} \frac{d^{2}M_{Z}^{L}}{d\psi_{L}{}^{2}}=\frac{d^{2}M_{Z}^{P}}{d\psi_{L}{}^{2}}+\left[\left(\frac{d^{2}F_{Y}^{P}}{d\psi_{L}{}^{2}}-2\frac{dF_{X}^{P}}{d\psi_{L}}-F_{Y}^{P}\right)x_{P}+\left(\frac{d^{2}F_{X}^{P}}{d\psi_{L}{}^{2}}-2\frac{dF_{Y}^{P}}{d\psi_{L}}-F_{X}^{P}\right)y_{P}\right]\cos\psi_{L}\\ -\left[\left(2\frac{dF_{Y}^{P}}{d\psi_{L}}-F_{X}^{P}+\frac{d^{2}F_{X}^{P}}{d\psi_{L}{}^{2}}\right)x_{P}+\left(2\frac{dF_{X}^{P}}{d\psi_{L}}-F_{Y}^{P}+\frac{d^{2}F_{Y}^{P}}{d\psi_{L}{}^{2}}\right)y_{P}\right]\sin\psi_{L} \end{array}$$

in which:

(93) $$\frac{d^{2}F_{X}^{P}}{d\psi_{L}{}^{2}}=\ddot{F_{X}^{P}}-\frac{\dot{F_{X}^{P}}}{\dot{\psi_{L}}}\;\ddot{\psi_{L}};\frac{d^{2}F_{Y}^{P}}{d\psi_{L}{}^{2}}=\ddot{F_{Y}^{P}}-\frac{\dot{F_{Y}^{P}}}{\dot{\psi_{L}}}\;\ddot{\psi_{L}};\frac{d^{2}M_{Z}^{P}}{d\psi_{L}{}^{2}}=\ddot{M_{Z}^{P}}-\frac{\dot{M_{Z}^{P}}}{\dot{\psi_{L}}}\;\ddot{\psi_{L}}$$

For the case where only a concentrated moment is applied, the analytical expression of the inflection circle is reported Appendix D.

Following the flow of the work above, it is possible to find the analytical description of many other geometric loci important for kinematic synthesis, including the second Bresse’s circle, the centre of accelerations, the cubic of stationary curvature, the Burmester points, etc.

## 4. Numerical Examples and Experimental Evidence

In this section, some numerical applications of the analytical results obtained in Section 2 and Section 3 are shown. If the length, bending stiffness and loads are given, the only unknown is the angle $\psi_{L}$. The latter needs to be obtained using an attempt method with Equation (54). A fast method to address this issue is the bisection algorithm [31,33,45], which requires an interval search $\psi_{L}\in \left[\psi_{L1},\psi_{L2}\right]$ (the interval can be chosen to be very wide, e.g., (0,2π), to satisfy any load and configuration conditions). The error tolerance of the end angle is set to $10^{-8}$ in the following examples.

The kinematics of a flexure hinge connecting two rigid bodies, constrained and mobile, were examined. The material of the flexure hinge was ABS with E=2.3 GPa, the length was L=30 mm and the constant section was rectangular, 1 mm thick and 3 mm wide. The load was applied to the mobile rigid body at $x_{P}=5\;\mathrm{mm}$.

**Figure 6:**
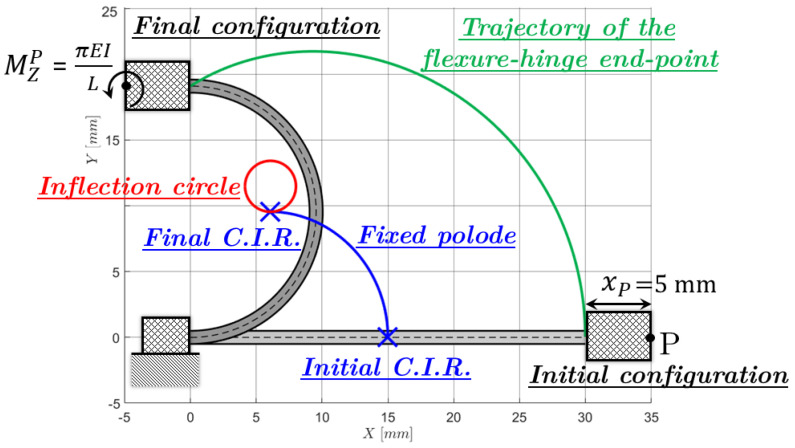
Flexure hinge loaded by a concentrated moment applied to the mobile rigid body.

The first case (Figure 6) concerns a straight flexure hinge, where a pure moment acts on the mobile rigid body. A fully analytical solution for this situation is reported in Appendix D. The applied moment is equal to $M_{Z}^{P}=\pi EI/L$, obtaining a final angle $\psi_{L}=\pi$. Figure 6 shows the trajectory of the endpoint and c.i.r. (i.e., the fixed polode) and the inflection circle computed in the final configuration. This is a special case, inasmuch as the presence of only a concentrated moment guarantees that the polode is conservative, i.e., loading-path independent. It is possible to observe that the initial position of the c.i.r. coincides with the centre of the flexure hinge; however, it moves away during the configuration change out of the flexure hinge axis and closer to the fixed body. For this reason, the pseudo-rigid body approach used in Howell’s simplest version [2], which involves a single lumped hinge in the middle of the flexure hinge, causes a significant error in the predicted motion [46].

**Figure 7:**
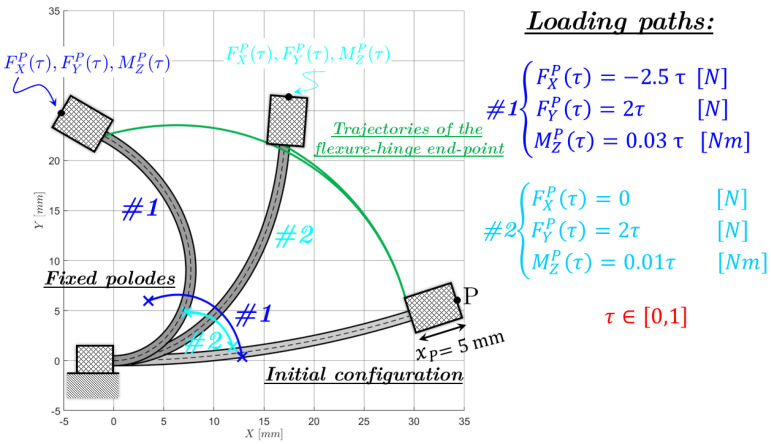
Flexure-hinge loaded by two different loading paths.

A less trivial example is shown in Figure 7. In this case, an initially curved flexure hinge ($\vartheta^{\prime }=10{\;m}^{-1}$) is connected to a rigid body loaded with both forces and a moment. The two loading paths (detailed in Figure 7) have a linear trend but two different final loads.

As expected, for two different final loads, the two fixed polodes and therefore all the instantaneous invariants differ (all being dependent on the location of the c.i.r.).

**Figure 8:**
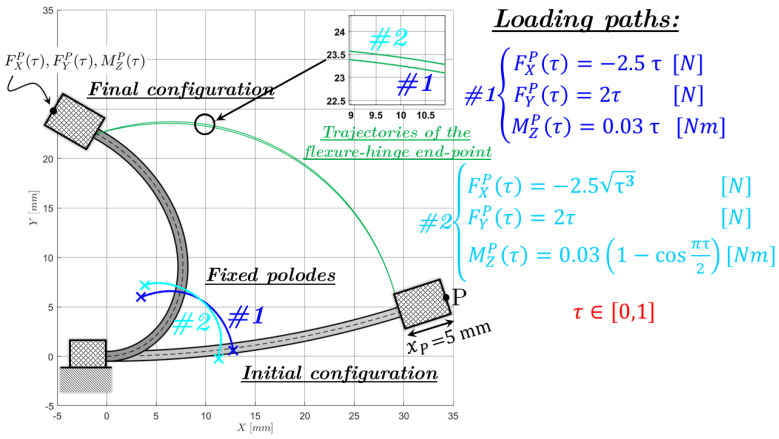
Flexure-hinge loaded by two loading paths with the same final loads but different rate-trends.

The case in Figure 8 examines the influence of the loading trend on the c.i.r. locations, keeping the same final loads. The first loading path is the same as above, while the second achieves the same final loads; however, they grow in a nonlinear way.

The two fixed polodes differ, as is evident in Figure 8. It should be noted that the initial positions of the c.i.r. do not coincide either.

This result is less intuitive than the previous one; however, it proves that, generally, instantaneous invariants are not conservative for compliant mechanisms.

Therefore, it is not possible to foresee the motion and features (i.e., instantaneous invariants) of a flexible mechanism if the dynamic knowledge of all acting loads is unknown. Indeed, in the examples presented above, the c.i.r. locations differ remarkably. In other words, one should be very careful to address the kinematic synthesis of compliant mechanisms with the same method used for rigid mechanisms connected through kinematic pairs.

**Figure 9:**
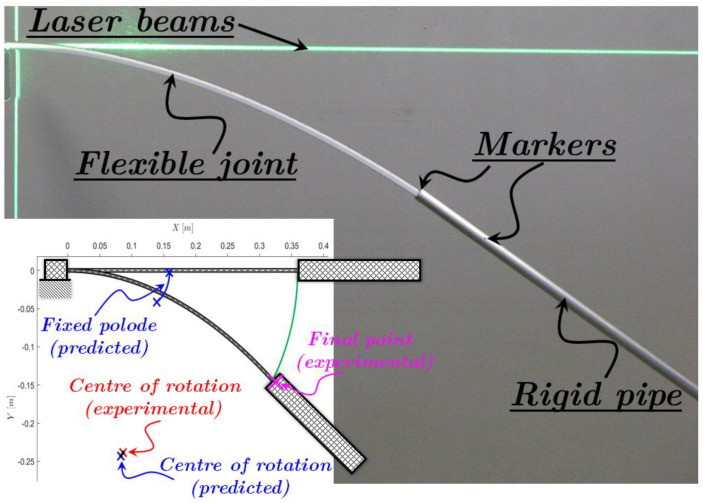
Picture of the experimental setup with added notations.

Some experiments were conducted on a flexible PVC beam constrained with two almost rigid pipes at the ends; one is fixed and the other free, subjected to gravity, as shown in Figure 9. The bending stiffness of the flexible beam was estimated through material testing and section measurement. The extrapolation of experimental data was conducted through a digital image analysis where the alignment of the instruments was assessed using laser beams. The experimental measurements were used to validate the method proposed in this paper. Comparisons between experimental evidence and numerical predictions were carried out to compare the overall (i.e., between the initial and final configuration) centre of rotation, as shown in Figure 9 (first row of Table 1). The two centres of rotation were very close, demonstrating that the method allows for the correct analysis of considerable displacements. It is interesting to note that the trajectory of the c.i.r. (i.e., the fixed polode) during the motion is not predictable a priori with only knowledge of the initial and final configurations, and it is mandatory to perform a reliable kinematic analysis. In Table 1, four cases are examined according to the length of the flexible joint, where the experimental and predicted coordinates of the centre of rotation and final angles are reported. All the results showed good agreement with small or relevant displacements; however, the error increased inversely with length, probably due to a lower precision of data acquisition when smaller displacements occur.

## 5. Conclusions

This paper investigated the elasto-kinematics and the kinematic features of motion (i.e., instantaneous invariants) of compliant mechanisms based on flexure hinges. A comprehensive deduction of the differential equation that governs the nonlinear geometric behaviour of flexure hinges was presented. These equations were analytically addressed, assuming the extensional and shear strains to be negligible, the section and the initial curvature constant and the distributed loads null. The analytical solution provides a remarkable computational advantage compared to numerical methods (e.g., Runge–Kutta); it allows for the management of a single point of interest (e.g., the extreme of the flexure hinge), avoiding a full-length integration. This feature is crucial to deduce the analytical expressions of the instantaneous invariants that require the derivatives of the endpoint of the flexure hinge. Two instantaneous invariants were investigated, the centre of instantaneous rotation and the inflection circle (first Bresse’s circle). The main obtained result is that the c.i.r. locations (i.e., fixed polode) are not conservative, i.e., they depend on the loading path and evolution. Therefore, all the other instantaneous invariants are not conservative; as a consequence, the notion of instantaneous geometric invariants (i.e., independent of the motion time law) decays.

These results were numerically verified in some examples, and a simple experimental validation was conducted using optical means with the aim of verifying that the step-by-step analysis resulted in the final configuration experienced.

The obtained equation, although given for flexure hinges with a constant section, may be extended to notched flexure hinges given that the main deformation is due to the central part presenting a constant section. Furthermore, the achieved results could open new avenues to define the Jacobian constraint matrix (used in multibody codes) of flexure hinges, where it should appear not only as a function of the geometry and material properties but also of the actual loads.

## Figures and Tables

**Table 1:** Experimental and predicted position of rotation centre and final end angle for four lengths of flexible joint.

Flexible Joint Lengths [mm]	Experimental Centre of Rotation [mm]	Predicted Centre of Rotation [mm]	Experimental Final Angle [deg]	Predicted Final Angle [deg]
360	(87.3, −238.6)	(83.7, −242.9)	41.6	42.7
270	(85.2, −169.4)	(85.3, −168.2)	33.0	32.6
180	(70.7, −100.1)	(71.4, −97.4)	22.2	21.5
90	(40.3, −47.1)	(41.5, −38.9)	12.4	10.2

## Data Availability

Not applicable.

## Appendix A. Proof of the Skewness of the Curvature Tensor

The change-of-basis Λ is an orthogonal tensor:

(A1) $$\Lambda {\left(\Lambda \right)}^{T}=I$$

Applying the derivative with respect to s of Equation (A1), the following is obtained:

(A2) $$\frac{\partial \Lambda }{\partial s}{\left(\Lambda \right)}^{T}=-\Lambda \frac{\partial {\left(\Lambda \right)}^{T}}{\partial s}=-{\left[\frac{\partial \Lambda }{\partial s}{\left(\Lambda \right)}^{T}\right]}^{T}\;$$

The above is the definition of a skew-symmetric tensor. Therefore, the curvature tensors in Equations (7) and (8) are skew-symmetric.

## Appendix B. Other Relations Regarding the Equilibrium

The forces and moment F(s),M(s) applied at the generic curvilinear abscissa s can also be expressed as functions of the applied loads $F_{L},M_{L}$ at s=L, as well as the distributed loads:

(A3) $$F_{X}\left(s\right)=F_{X}^{L}+\overset{L}{\underset{s}{\int }}q_{X}\left(\tilde{s}\right)\;d\tilde{s}$$

(A4) $$F_{Y}\left(s\right)=F_{Y}^{L}+\overset{L}{\underset{s}{\int }}q_{Y}\left(\tilde{s}\right)\;d\tilde{s}$$

(A5) $$\begin{array}{c} M_{Z}\left(s\right)=M_{Z}^{L}-\left(Y_{L}-Y\right)F_{X}^{L}+\left(x_{L}-x\right)F_{Y}^{L}\\ \;\;\;\;\;\;+\int_{s}^{L}\left[-\left(\tilde{Y}-Y\right)q_{X}\left(\tilde{s}\right)-\left(\tilde{X}-X\right)q_{Y}\left(\tilde{s}\right)+m_{Z}\left(\tilde{s}\right)\right]\;d\tilde{s} \end{array}$$

where $X_{L}=X\left(s=L\right)\;,\;Y_{L}=Y\left(s=L\right)$ are the coordinates of the point in s=L.

By applying the moment equilibrium with the pole at s=0, the components of $F_{0},M_{0}$ can be expressed as function of $F_{L},M_{L}$:

(A6) $$F_{X}^{0}=-F_{X}^{L}-\overset{L}{\underset{0}{\int }}q_{X}\left(s\right)\;ds$$

(A7) $$F_{Y}^{0}=-F_{Y}^{L}-\overset{L}{\underset{0}{\int }}q_{Y}\left(s\right)\;ds$$

(A8) $$M_{Z}^{0}=-M_{Z}^{L}+Y_{L}F_{X}^{L}-X_{L}F_{Y}^{L}+\overset{L}{\underset{0}{\int }}\left[Y\;q_{X}\left(s\right)-X\;q_{Y}\left(s\right)-m_{Z}\left(s\right)\right]\;ds$$

On the contrary, by applying the moment equilibrium with the pole at s=L, the components of $F_{L},M_{L}$ can be expressed as function of $F_{0},M_{0}$:

(A9) $$F_{X}^{L}=-F_{X}^{0}-\overset{L}{\underset{0}{\int }}q_{X}\left(s\right)\;ds$$

(A10) $$F_{Y}^{L}=-F_{Y}^{0}-\overset{L}{\underset{0}{\int }}q_{Y}\left(s\right)\;ds$$

(A11) $$\begin{array}{l} M_{Z}^{L}=-M_{Z}^{0}-Y_{L}F_{X}^{0}+X_{L}F_{Y}^{0}\\ +\int_{0}^{L}\left[-\left(Y_{L}-Y\right)q_{X}\left(s\right)-\left(X_{L}-X\right)q_{Y}\left(s\right)-m_{Z}\left(s\right)\right]\;ds \end{array}$$

## Appendix C. A Useful Trick to Avoid the Singularities of Some Integrals

In the presence of an inflection point, the denominator of the Equations (42)–(44) becomes null for $\psi \left(s\right)=\psi_{in}$. If an internal inflection point occurs at $\psi \left(s\right)=\psi_{i}$, the integral of Equation (42) can be separated into two components using Equation (41):

(A12) $$L=\overset{\psi_{in}}{\underset{\psi_{0}}{\int }}\frac{sign\left(\psi^{\prime }\right)}{f\left(\psi \right)}d\psi +\overset{\psi_{L}}{\underset{\psi_{in}}{\int }}\frac{sign\left(\psi^{\prime }\right)}{f\left(\psi \right)}d\psi$$

The previous integrand function becomes singular for $\psi_{i}$. Therefore, to overcome this problem [76], we introduce a very small positive quantity ϵ≪1 (numerically, $\epsilon ≅10^{-4}$ is sufficient), such that:

(A13) $$L=sign\left(\psi_{0}^{\prime }\right)\left[\overset{\psi_{i}-\epsilon }{\underset{\psi_{0}}{\int }}\frac{\;d\psi }{f\left(\psi \right)}+\overset{\psi_{in}}{\underset{\psi_{in}-\epsilon }{\int }}\frac{\;d\psi }{f\left(\psi \right)}\right]+sign\left(\psi_{L}^{\prime }\right)\left[\overset{\psi_{L}}{\underset{\psi_{in}+\epsilon }{\int }}\frac{\;d\psi }{f\left(\psi \right)}+\overset{\psi_{in}+\epsilon }{\underset{\psi_{in}}{\int }}\frac{\;d\psi }{f\left(\psi \right)}\right]$$

The two integrals with extremes of integration $\left[\psi_{in}-\epsilon ,\;\psi_{in}\right]$ and $\left[\psi_{in},\;\psi_{in}+\epsilon \right]$, by virtue of the small value of ϵ, can be linearized (and then integrated) using the change in variables $\psi =\psi_{in}-\omega$, which implies ω∈[0,ϵ], obtaining:

(A14) $$f\left(\psi \right)=f\left(\psi_{in}-\omega \right)≅\sqrt{\frac{2}{EI}\left[c-F_{X}\left(\cos\psi_{in}+\omega \sin\psi_{in}\right)-F_{Y}\left(\sin\psi_{in}-\omega \cos\psi_{in}\right)\right]}$$

Hence:

(A15) $$I_{1}\left(\epsilon \right)=\overset{\psi_{in}}{\underset{\psi_{in}-\epsilon }{\int }}\frac{\;d\psi }{f\left(\psi \right)}=\overset{\epsilon }{\underset{0}{\int }}\frac{\;d\omega }{f\left(\psi_{in}-\omega \right)}=\sqrt{2EI}\;\frac{\sqrt{\tilde{A}+\epsilon \tilde{B}}-\sqrt{\tilde{A}}}{\tilde{B}}$$

(A16) $$I_{2}\left(\epsilon \right)=\overset{\psi_{in}+\epsilon }{\underset{\psi_{in}}{\int }}\frac{\;d\psi }{f\left(\psi \right)}=\overset{0}{\underset{-\epsilon }{\int }}\frac{\;d\omega }{f\left(\psi_{in}-\omega \right)}=\sqrt{2EI}\;\frac{\sqrt{\tilde{A}}-\sqrt{\tilde{A}-\epsilon \tilde{B}}}{\tilde{B}}$$

where:

(A17) $$\tilde{A}=c-F_{X}\cos\psi_{in}-F_{Y}\sin\psi_{in}$$

(A18) $$\tilde{B}=F_{Y}\cos\psi_{in}-F_{X}\sin\psi_{in}$$

Therefore, the integral of Equation (43) (or Equation (A12)) in the presence of an inflection point is:

(A19) $$L=sign\left(\psi_{0}^{\prime }\right)\left[\overset{\psi_{in}-\epsilon }{\underset{\psi_{0}}{\int }}\frac{\;d\psi }{f\left(\psi \right)}+I_{1}\right]+sign\left(\psi_{L}^{\prime }\right)\left[\overset{\psi_{L}}{\underset{\psi_{in}+\epsilon }{\int }}\frac{\;d\psi }{f\left(\psi \right)}+I_{2}\right]$$

and the singularity no longer appears.

The same trick can be applied to the integrals in Equations (44) and (45), obtaining:

(A20) $$X\left(\psi \right)=X_{0}+sign\left(\psi_{0}^{\prime }\right)\left[\overset{\psi_{in}-\epsilon }{\underset{\psi_{0}}{\int }}\frac{\;\cos\tilde{\psi }}{f\left(\tilde{\psi }\right)}d\tilde{\psi }+I_{3}\right]+$$

(A21) $$\begin{array}{c} \;\;\;\;\;\;\;\;\;\;+sign\left(\psi_{L}^{\prime }\right)\left[\overset{\psi }{\underset{\psi_{in}+\epsilon }{\int }}\frac{\cos\tilde{\psi }}{f\left(\tilde{\psi }\right)}d\tilde{\psi }+I_{4}\right]\\ Y\left(\psi \right)=y_{0}+sign\left(\psi_{0}^{\prime }\right)\left[\int_{\psi_{0}}^{\psi_{in}-\epsilon }\frac{\;\sin\tilde{\psi }}{f\left(\tilde{\psi }\right)}d\tilde{\psi }+I_{5}\right]+\\ \;\;\;\;\;\;\;\;\;\;+sign\left(\psi_{L}^{\prime }\right)\left[\int_{\psi_{in}+\epsilon }^{\psi }\frac{\sin\tilde{\psi }}{f\left(\tilde{\psi }\right)}d\tilde{\psi }+I_{6}\right] \end{array}$$

where:

(A22) $$I_{3}\left(\epsilon \right)=\frac{I_{1}\left(\epsilon \right)\cos\psi_{in}}{sign\left(\psi_{L}^{\prime }\right)}+\frac{2\sin\psi_{in}}{3{\tilde{B}}^{2}}\left[\left(\epsilon \tilde{B}-2\tilde{A}\right)\sqrt{\tilde{A}+\epsilon \tilde{B}}+2\tilde{A}\sqrt{\tilde{A}}\right]$$

(A23) $$I_{4}\left(\epsilon \right)=\frac{I_{2}\left(\epsilon \right)\cos\psi_{in}}{sign\left(\psi_{L}^{\prime }\right)}+\frac{2\sin\psi_{in}}{3{\tilde{B}}^{2}}\left[\left(\epsilon \tilde{B}+2\tilde{A}\right)\sqrt{\tilde{A}-\epsilon \tilde{B}}-2\tilde{A}\sqrt{\tilde{A}}\right]$$

(A24) $$I_{5}\left(\epsilon \right)=\frac{I_{1}\left(\epsilon \right)\sin\psi_{in}}{sign\left(\psi_{L}^{\prime }\right)}+\frac{2\cos\psi_{in}}{3{\tilde{B}}^{2}}\left[\left(\epsilon \tilde{B}-2\tilde{A}\right)\sqrt{\tilde{A}+\epsilon \tilde{B}}+2\tilde{A}\sqrt{\tilde{A}}\right]$$

(A25) $$I_{6}\left(\epsilon \right)=\frac{I_{2}\left(\epsilon \right)\sin\psi_{in}}{sign\left(\psi_{L}^{\prime }\right)}+\frac{2\cos\psi_{in}}{3{\tilde{B}}^{2}}\left[\left(\epsilon \tilde{B}+2\tilde{A}\right)\sqrt{\tilde{A}-\epsilon \tilde{B}}-2\tilde{A}\sqrt{\tilde{A}}\right]$$

It is worth pointing out that Equations (A13), (A20) and (A21) hold even if an inflection point happens at one end (e.g., a cantilever beam loaded by a concentrated force at the end), simply considering that $\psi_{in}=\psi_{0}$ or $\psi_{in}=\psi_{L}$.

## Appendix D. Fully Analytical Solution of Polodes (Fixed and Mobile) and Inflection Circle for a Flexure Hinge Loaded by a Concentrated Moment

Taking into account the assumptions of Section 2.1, the deformed configuration of a flexure hinge loaded only by a concentrated moment $M_{Z}^{P}$ is represented by the following parametric equations [53]:

(A26) $$\psi \left(s\right)=\psi_{0}+\left(\vartheta^{\prime }+\frac{M_{Z}^{P}}{EI}\right)s$$

(A27) $$X\left(s\right)=X_{0}+\frac{\sin\left[\left(\vartheta^{\prime }+\frac{M_{Z}^{P}}{EI}\right)s\right]}{\left(\vartheta^{\prime }+\frac{M_{Z}^{P}}{EI}\right)}\;$$

(A28) $$Y\left(s\right)=Y_{0}+\frac{1-\cos\left[\left(\vartheta^{\prime }+\frac{M_{Z}^{P}}{EI}\right)s\right]}{\left(\vartheta^{\prime }+\frac{M_{Z}^{P}}{EI}\right)}$$

From the latter equations, the terms $X_{L},Y_{L},\frac{dX_{L}}{d\psi_{L}},\frac{dY_{L}}{d\psi_{L}},\frac{d^{2}X_{L}}{d\psi_{L}{}^{2}},\frac{d^{2}Y_{L}}{d\psi_{L}{}^{2}}$ that form the parametric equations of the fixed and mobile polodes in Equations (55), (56), (58) and (59) and the inflection circle in Equations (74)–(77) can be made explicit. Setting $\psi_{0}=X_{0}=Y_{0}=0$ for the sake of clarity, one obtains:

(A29) $$\psi_{L}=\left(\vartheta^{\prime }+\frac{M_{Z}^{P}}{EI}\right)L$$

(A30) $$X_{L}=\frac{L\;\sin\psi_{L}}{\psi_{L}}\;\;\;\;\;\;\;$$

(A31) $$Y_{L}=\frac{L\;\left(1-\cos\psi_{L}\right)}{\psi_{L}}$$

(A32) $$\frac{dX_{L}}{d\psi_{L}}=\frac{L-X_{L}}{\psi_{L}\;}-Y_{L}\;\;$$

(A33) $$\frac{dY_{L}}{d\psi_{L}}=X_{L}-\frac{Y_{L}}{\psi_{L}}\;\;\;\;\;\;$$

(A34) $$\frac{d^{2}X_{L}}{d\psi_{L}{}^{2}}=\frac{X_{L}-L}{\psi_{L}{}^{2}}-\frac{dY_{L}}{d\psi_{L}}-\frac{1}{\psi_{L}}\frac{dX_{L}}{d\psi_{L}}$$

(A35) $$\frac{d^{2}Y_{L}}{d\psi_{L}{}^{2}}=\frac{dX_{L}}{d\psi_{L}}-\frac{1}{\psi_{L}}\frac{dY_{L}}{d\psi_{L}}+\frac{Y_{L}}{\psi_{L}{}^{2}}$$
